# Stress Response of *Siniperca chuatsi* to Transport Stimuli Using Compound Feed and Live Bait

**DOI:** 10.3390/ani15142154

**Published:** 2025-07-21

**Authors:** Yuanliang Duan, Qiang Li, Zhipeng Huang, Zhongmeng Zhao, Han Zhao, Yang Feng, Senyue Liu, Chengyan Mou, Jian Zhou, Lu Zhang

**Affiliations:** 1 Fisheries Research Institute, Sichuan Academy of Agricultural Sciences (Sichuan Fisheries Research Institute), Chengdu 611731, China; 2 College of Fisheries, Huazhong Agricultural University, Wuhan 430070, China

**Keywords:** *Siniperca chuatsi*, stress response, compound feed, antioxidant capacity, transport stimuli

## Abstract

The transition from live bait (LF) feeding to compound feed (CF) feeding in aquaculture is of great production significance. In recent decades, cultivation with CF has become a focus for practitioners and researchers dealing with *Siniperca chuatsi*. This study focused on experimental subjects of *S. chuatsi* fed with CF and LF, using short-distance transportation as a stimulating factor. For the first time, the differences between *S. chuatsi* fed with CF and LF were analyzed from the perspective of stress response during transportation. This study found that after transportation stimulation, the activities of LZM and the contents of MDA, TGs, and glucose in the brain, liver, kidneys, muscles, stomach, pyloric caecum, intestines, and blood of *S. chuatsi* fed with CF were higher compared to *S. chuatsi* fed with LF (*p* < 0.05). Significant differences were observed in the impacts of various diets on the gastrointestinal tract, particularly in the intestine. In summary, this study found that *S. chuatsi* fed with CF could retain more energy after transport stimulation and exhibited stronger resistance to microbial stress, but they had a weaker antioxidant capacity. Therefore, in future research on CF for *S. chuatsi*, we need to focus on its ability to enhance antioxidant capacity.

## Simple Summary

This study addresses the challenge of transitioning *Siniperca chuatsi* (mandarin fish) from live bait (LF) to compound feed (CF) in aquaculture by examining their stress responses during short-distance transportation. The aim was to compare the physiological and biochemical differences between CF-fed and LF-fed fish under transport stress. The results showed that CF-fed fish exhibited higher levels of lysozyme (LZM) activity, malondialdehyde (MDA), triglycerides (TGs), and glucose in multiple tissues (brain, liver, kidneys, muscles, stomach, pyloric caecum, intestines, and blood) compared to LF-fed fish (*p* < 0.05), indicating greater energy retention and microbial resistance but weaker antioxidant capacity. The intestine was particularly affected by dietary differences. This study concludes that while CF-fed *S. chuatsi* demonstrate benefits in energy metabolism and microbial defense, their reduced antioxidant capacity highlights the need for future research to improve CF formulations to enhance oxidative stress resistance. These findings are valuable for optimizing aquaculture practices, ensuring healthier fish production, and supporting sustainable feed development in the industry.

## 1. Introduction

The mandarin fish (*Siniperca chuatsi*) belongs to the Perciformes, Serranidae, and *Siniperca*. It is mainly distributed in East Asia and is considered a high-quality aquatic product in China due to its delicate meat, lack of muscle spines, delicious taste, and high nutritional value [1]. According to the 2024 *China Fisheries Statistical Yearbook*, the national production of *S. chuatsi* aquaculture in 2023 was 477,592 tons [2], making it one of the most promising specialty freshwater fish aquaculture varieties. When the *S. chuatsi* fry start feeding, they only accept live prey and refuse dead prey or artificial feed [3]. In the 1980s and 1990s, Chinese scholars conducted research on the feeding behavior of *S. chuatsi* [4,5] and, based on this, established the conditional reflex transition method [6,7,8]. This method begins by initially providing live bait fish. Once a strong feeding response is established, the next step is to switch to dead bait fish and bait fish blocks. Following this, surimi feed is introduced, with a gradual reduction in fish meat content, ultimately transitioning to artificial compound feed. Typically, this process takes about five days, although it can be extended based on the specific circumstances. This method initially mastered the technique of domesticating feed for *S. chuatsi* and achieved a breakthrough in their active consumption of compound feed (CF). The domestication process of *S. chuatsi* is laborious and challenging to manage. However, given the inadequate supply of bait fish and the susceptibility to pathogenic bacteria in LF, it is imperative for the *S. chuatsi* breeding industry to transition from LF breeding to artificial mixed-feed breeding.

Transportation is a crucial aspect of aquaculture operations. Before the fry enter the aquaculture farm and the adult fish enter the sales market, they generally need to undergo a certain period of transportation. Transportation can induce stress reactions in fish, which may be attributed to factors such as vibrations, crowding, ammonia nitrogen, and temperature [9]. The water vibrations caused by various uncontrollable factors during transportation can trigger stress reactions in fish, such as an increase in plasma cortisol [10,11]. Fish require a reasonable density during transportation. If the density is too high, it may lead to crowding stress and reduce the survival rate of the fish [12]. Crowding stress may also lead to an increase in cortisol content in fish plasma [13] and may even result in a decrease in muscle hardness and cohesion in fish [14]. During transportation, the accumulation of ammonia nitrogen in the water is a crucial factor affecting the survival rate of fish as they continue to metabolize. Acute ammonia nitrogen stress can cause significant damage to the brain, gills, liver, and other tissue structures of fish, reduce the body’s immunity, and even threaten life [15,16]. Fish are exposed to high-temperature conditions, which lead to enhanced metabolism and increased oxygen consumption. Therefore, cooling measures are undertaken during transportation to maintain the vitality of the products. If the temperature rises, it will have a negative impact on the liver function [17], immune function [18,19], and meat quality [20,21] of the fish.

Ensuring the safety and high quality of fish, improving their survival rate, increasing their transportation volume, extending the transportation time, and reducing transportation costs are the focal points for researchers and industries. To create these hotspots, the key lies in transportation strategies and the physical fitness of fish bodies. Today, the transportation methods for fish survival are mainly divided into water transportation and waterless transportation [22]. The former is further divided into bagging/boxing closed transportation, circulating-water closed transportation, bagging/boxing open transportation, and circulating-water open transportation. The latter is further divided into anesthetic/ecological ice-temperature anesthesia transportation. Among these transportation methods, physical improvement and temporary culture before transportation [23,24,25,26], anesthesia during transportation [27], temperature control, oxygenation, and appropriate density [28,29] can all reduce transportation stress on fish and improve their survival rate during transportation. Compared with refrigerated or frozen fish products, the selling price of live fish products is relatively high. This price difference is particularly noticeable for economically valuable fish, where the price can typically be 2–5 times higher [22]. At present, research on transportation for fish survival, both domestically and internationally, focuses on technological breakthroughs. However, there has been relatively little research on the stress response, physiological and biochemical characteristics, and muscle quality changes of fish in these technological applications. This makes it difficult to identify precise targets for monitoring and controlling the process of fish survival and transportation. Therefore, to ensure the survival and quality of fish during transportation, it is crucial to conduct research and develop transportation strategies, key technologies, and explore the physiological status of the fish themselves.

In this study, we used *S. chuatsi* with CF and LF as experimental subjects, with short-distance transportation as a stimulating factor. For the first time, the dynamic changes in stress substances in various tissues were utilized to analyze the stress response of *S. chuatsi* raised on different diets to transportation stimuli. The aim of this study was to investigate the antioxidant enzyme activities, MDA levels, and glucose concentrations of *S. chuatsi* after transportation, and to elucidate the physical conditions of *S. chuatsi* raised with different diets from a physiological perspective. This would provide some data support for further research on the transformation of *S. chuatsi* from LF breeding to CF breeding.

## 2. Materials and Methods

### 2.1. Source of Experimental Organisms, and Experimental Design

This study collected a total of 60 *S. chuatsi* fed with CF (mean length, 25.88 ± 0.54 cm; mean weight, 254.11 ± 10.30 g) and 55 *S. chuatsi* fed with live bait (LF, mean length, 24.68 ± 1.48 cm; mean weight, 244.69 ± 1.39 g), which were farmed at the Institute of Fisheries, Sichuan Academy of Agricultural Sciences (Sichuan Fisheries Research Institute) (Chengdu, China). Before the *S. chuatsi* were randomly caught, they were fed various types of feed for two months. The protein content of the compound feed was 40%, while that of the live feed was 19%. The *S. chuatsi* were packed separately in nylon mesh cages and temporarily cultured in the same indoor breeding pool with a water temperature of 15 °C. The temporary culture period was 48 h without feeding.

After the temporary culture, six fish were randomly collected, and their brains, livers, kidneys, muscles, stomachs, pyloric caeca, intestines, and blood were collected under low-temperature conditions. At the same time, 10 fish fed with CF and 10 fish fed with LF were randomly collected and placed in the same water tank. The stocking density was 10.39 g/L, but they were separated by nylon mesh. The water used for transportation came from a temporary pond (480 L), with a transportation time of 2.5 h, and the oxygenation equipment was activated throughout the entire process. The water quality parameters before and after transportation are shown in Table S1. Immediately after the transportation was completed, 6 fish were randomly collected from each treatment group. The brains, livers, kidneys, muscles, stomachs, pyloric caeca, intestines, and blood of *S. chuatsi* were collected under ice-bath conditions, where the anatomical discs containing the fish were placed on ice. All of the tissues were used to detect acid phosphatase (ACP), alkaline phosphatase (AKP), lactate dehydrogenase (LDH), total antioxidant capacity (T-AOC), catalase (CAT), superoxide dismutase (SOD), malondialdehyde (MDA), and lysozyme (LZM). Furthermore, blood was used to measure the levels of triglycerides (TGs) and glucose.

### 2.2. Tissue Sample Collection

The fish were euthanized with a high dose of tricaine methanesulfonate (250 mg/L, Aladdin, Shanghai, China) and disinfected with 75% alcohol. Firstly, blood was collected from the tail vein. Afterward, the fish were dissected, and their brains, livers, kidneys, muscles, stomachs, pyloric caeca, and intestines were collected. Then, 0.1 g of wet tissue samples was weighed, added to 0.9% normal saline at a ratio of 1:9 (W/V), and homogenized in an ice-water bath using a homogenizer. The mixture was then centrifuged at 1369× *g* for 10 min, and the supernatant was collected for further research. Blood samples were left overnight at 4 °C, centrifuged at 1369× *g* for 10 min, and the supernatant was collected for further research.

### 2.3. Enzymatic Activities Analysis

In this study, the activities or contents of ACP, AKP, LDH, T-AOC, CAT, SOD, MDA, LZM, TGs, and glucose were detected using the Acid Phosphatase Assay Kit, Alkaline Phosphatase Assay Kit, Lactate Dehydrogenase Assay Kit, Total Antioxidant Capacity Assay Kit, Catalase Assay Kit, Superoxide Dismutase Assay Kit, Malondialdehyde Assay Kit, Lysozyme Assay Kit, Triglyceride Assay Kit, and Glucose Assay Kit (Nanjing Jiancheng Bioengineering Institute, Nanjing, China). The detection steps were carried out according to the operating instructions of the reagent kit. In addition, the calculation formulae for these detection indicators are detailed in the Supplementary Materials.

### 2.4. Data Analysis

SPSS 19.0 and Excel software were used for statistical analysis, while the images were created using Adobe Photoshop CC 2018 and Excel software. The equality of variance testing and normality testing were performed using SPSS 19.0 before the formal analysis. The multiple comparisons and one-way analysis of variance were conducted; *p* ≤ 0.05 was considered statistically significant, and the results were presented as the mean ± standard deviation.

## 3. Results

### 3.1. Analysis of Stress Response Before Transportation

The results of stress-related indicators in *S. chuatsi* fed with CF and LF before transportation are presented in Tables S1 and S2. Before transportation, the physiological status of *S. chuatsi* was generally consistent, and most of the stress indicators detected did not reach a significant level. Only the following indicators reached significance: MDA (*p* = 0.025) content in brain tissues; ACP (*p* = 0.013) and LDH (*p* = 0.020) activities and MDA (*p* = 0.017) content in liver tissues; ACP and AKP activities in kidney (*p* = 0.000 and *p* = 0.015) and muscle tissues (*p* = 0.000 and *p* = 0.010), respectively; ACP (*p* = 0.000) and AKP (*p* = 0.015) activities in kidney tissue; and ACP activities in pyloric caecum (*p* = 0.000) and intestine tissues (*p* = 0.000).

### 3.2. Analysis of Stress Response After Transportation

**Figure 1:**
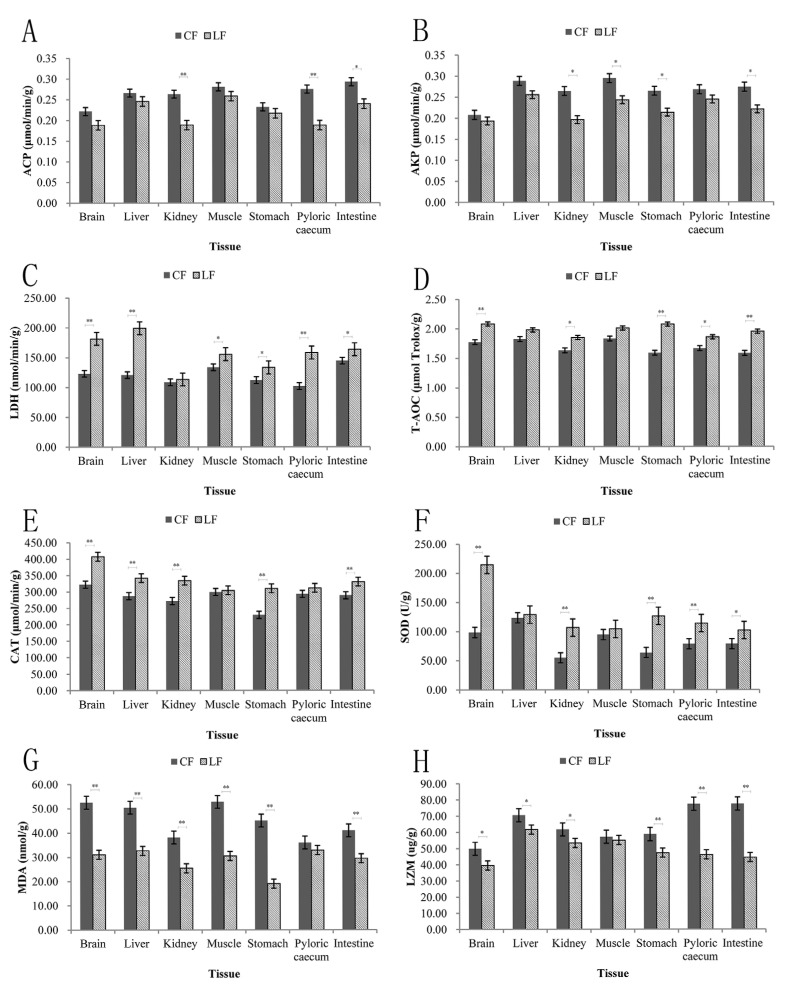
The stress response results of enzyme activities. (**A**–**H**) represent ACP, AKP, LDH, T-AOC, CAT, SOD, MDA and LZM, respectively. CF represents the *S. chuatsi* fed with compound feed. LF represents the *S. chuatsi* fed with live bait. The sample is wet weight. * and ** represent *p* < 0.05 and *p* < 0.01.

The stress response results of enzyme activities are shown in Figure 1. The activities of ACP, AKP, and LZM, as well as the contents of MDA in all tissues, were higher in the CF group than in the LF group. Conversely, the activities of LDH, T-AOC, CAT, and SOD were lower in the CF group compared to the LF group. Significant differences in activities were observed in the kidneys, pyloric caecum, and intestines for ACP. For AKP, significant differences in activities were observed in the kidneys, muscle, stomach, and intestines. Only the activity difference in the kidneys for LDH did not reach a significant level. T-AOC showed significant differences in activities in the brain, kidneys, stomach, pyloric caecum, and intestines. CAT exhibited significant differences in activities in the brain, liver, kidneys, stomach, and intestines. SOD displayed significant differences in activities in the brain, kidneys, stomach, pyloric caecum, and intestines. MDA showed that only its activity difference in the pyloric caecum did not reach a significant level. For LZM, only the difference in its activity in the muscle did not reach a significant level.

Using LF as a reference, we conducted correlation analysis based on relevant indicators that reached a significant level. The activities of LHD, T-AOC, CAT, and SOD in brain tissue were decreased, while MDA and LZM were increased. The activities of LDH and CAT in liver tissue decreased, while MDA and LZM increased. The activities of T-AOC, CAT, and SOD in kidney tissue were decreased, while ACP, AKP, MDA, and LZM were increased. The activity of LDH in muscle tissue was decreased, while AKP and MDA levels were increased. The activities of LHD, T-AOC, CAT, and SOD in stomach tissue were decreased, while AKP, MDA, and LZM were increased. The activities of LDH, T-AOC, and SOD in the pyloric caecum tissue were decreased, while ACP and LZM were increased. The activities of LHD, T-AOC, CAT, and SOD in intestinal tissue were decreased, while ACP, AKP, MDA, and LZM were increased.

In the blood, the differences in ACP, AKP, and T-AOC activities between CF and LF were minimal, as shown in Table 1. The activities of LDH, CAT, SOD, MDA, LZM, TGs, and glucose showed significant differences between CF and LF. Using LF as a reference, the activities of LDH, CAT, and SOD decreased, whereas MDA, LZM, TG, and glucose levels increased.

### 3.3. Analysis of Changes in Stress Response Before and After Transportation

In this study, the changes in stress-related indicators of *S. chuatsi* before and after experiencing transport stimuli were analyzed in terms of growth percentages, and the experimental results are presented in Table S3. The growth rate of ACP activities in the CF group was 14.32–32.71%, while that in the LF group was 7.57–22.31%. The growth rate of AKP activities in the CF group was 14.63−25.27%, while that in the LF group was 5.23–19.16%. The growth rate of LDH activities in the CF group was 28.63–63.75%, while that in the LF group was 62.23–106.36%. The growth rate of T-AOC activities in the CF group was 11.61–30.59%, while that in the LF group was 26.08–46.24%. The growth rate of CAT activities in the CF group was 18.90–92.88%, while that in the LF group was 44.81–192.94%. The growth rate of SOD activities in the CF group was 33.91–123.51%, while that in the LF group was 57.13–179.18%. The growth rate of MDA contents in the CF group was 102.35–228.57%, while that in the LF group was 31.37–135.01%. The growth rate of LZM activities in the CF group was 88.70–180.86%, while that in the LF group was 51.22–123.50%. The growth rate of TG content in the CF group was −18.15%, while that in the LF group was −35.79%. The growth rate of glucose content in the CF group was −15.74%, while in the LF group it was −31.61%. When the growth rates of stress-related indicators were emphasized without considering their relative weights, three indicators were found in the blood of *S. chuatsi* fed with CF, followed by two indicators in the intestines and muscles. *S. chuatsi* fed with LF exhibited two indicators each in the liver, stomach, and blood tissues.

## 4. Discussion

Ensuring the survival of fish during transportation has always been a primary concern for aquaculture workers and researchers. As research deepens, scientists have found that focusing solely on survival rates is no longer sufficient to meet the demands of production and life. Fish fry may die during subsequent aquaculture due to physical stress after transportation. Therefore, the physiological and biochemical status before and after transportation have gradually become the focus of researchers.

This study is the first worldwide investigation to explore the differences between *S. chuatsi* fed with CF and LF under transportation stimulation conditions. So far, there have been over 20 studies related to the transportation of *S. chuatsi*, a type of fish with high economic value. Most previous studies have introduced the transportation technology of *S. chuatsi* in the form of production guidance, with the main purpose of improving the survival rate of fish fry and adults. Cheng et al. [30,31] studied the effects of temperature and vibration frequency during transportation on *S. chuatsi*. They found that the optimal temperature for transportation was 10–15 °C, and that the vibration frequency should not exceed 50 Hz. Chen et al. [32,33] found that using a transportation method of carbon dioxide + oxygen anesthesia could prolong the survival time of *S. chuatsi*, reaching up to 23 h. Li et al. [34] discovered that malachite green and its metabolites were challenging to degrade during the transportation of *S. chuatsi*, and they suggested strengthening the supervision of malachite green in the transportation process to ensure the quality and safety of *S. chuatsi* aquatic products. In Cheng’s research [31], it was found that, after transportation, the respiratory rate of *S. chuatsi* significantly increased, blood glucose decreased, and LDH activities increased, mirroring the results of this study. In addition, the TG contents in this study also showed a decreasing trend, indicating that the metabolism of *S. chuatsi* was enhanced after undergoing transport stimulation, which is consistent with the results of Cheng [31]. In this study, in addition to an enhanced metabolism, the lipid oxidation, antioxidant capacity, and stress response to microorganisms in *S. chuatsi* were also improved. Due to the smaller amplitude of changes in ACP and AKP activities compared to enzyme activities such as T-AOC, SOD, and CAT, for *S. chuatsi*, it is necessary to prioritize addressing the oxidative damage to the fish body during transportation.

From initially being believed to only eat LF to now eating CF [3,35,36,37], *S. chuatsi* has undergone a complex domestication process, mainly aimed at improving its growth, quality, and yield. At present, research on the differences between *S. chuatsi* fed with CF and LF mainly focuses on the acceptability of CF and its impact on the growth performance and nutritional quality of *S. chuatsi*. Compared to *S. chuatsi* fed with LF, feeding it with CF can significantly increase the fatty acid contents of the fish body, such as eicosapentaenoic acid and docosahexaenoic acid [38]. Alam et al. [39] found that the protein content in the feed of bait fish was positively correlated with the growth rate and muscle protein content of bait fish and *S. chuatsi*, reaching a significant level. Feeding with CF can increase the amylase activity in the gastrointestinal tract of *S. chuatsi*, but it can inhibit the activities of gastric protease and intestinal trypsin, as well as the expression of small peptide transporter genes [40], thereby having adverse effects on the decomposition of feed protein and the absorption of small peptide transport. Some scholars have analyzed the gut microbiota of *S. chuatsi* fed with CF and LF [41] and subsequently supplemented with probiotics (*Bacillus*, *Lactobacillus*, *Clostridium*, etc.) to enhance CF feeding in *S. chuatsi*. A substantial body of research has shown that probiotics can enhance the tissue structure of the gut, boost digestive enzyme activity, and enhance the non-specific immune response of *S. chuatsi* [42,43,44]. Furthermore, probiotics can effectively address certain limitations associated with feeding CF to *S. chuatsi*, and this area may become a research hotspot in the future. In this study, compared to *S. chuatsi* fed with LF, *S. chuatsi* fed with CF had higher ACP and AKP activities before transportation (*p* < 0.05), which indicated that *S. chuatsi* fed with CF may have a stronger non-specific stress capacity. The final product of the lipid peroxidation reaction is MDA [45]. The MDA contents in the brain and liver tissues of *S. chuatsi* fed with CF were higher (*p* < 0.05), indicating that *S. chuatsi* fed with CF were more susceptible to membrane lipid oxidation. After transportation stimulation, the antioxidant enzyme activities of *S. chuatsi* fed with CF were inhibited compared to those fed with LF. This resulted in higher MDA contents and stronger LZM activities. Consequently, although the antioxidant capacity of *S. chuatsi* fed with CF was weakened, it exhibited a potential enhancement in innate immune markers. In addition, this study also found that, following environmental stimulation, the blood serves as a significant site for changes in stress-related indicators. When *S. chuatsi* fed with CF were stimulated, a notable stress response was observed in both the intestines and muscles. This response may be attributed to the fact that the muscles were subjected to increased transport stimuli, such as water waves, during the study. Additionally, the intestines may have experienced continuous stimulation from the CF that they consumed, with the transport stimuli further intensifying the pressure on the intestines. In compared to *S. chuatsi* fed with CF, those fed with LF exhibited stronger stimulation in their stomachs and blood, as well as heightened stimulation in their livers. This could be due to the fact that transportation stimuli accelerate the metabolism of *S. chuatsi*. Based on the significant differences between *S. chuatsi* fed with CF and LF, this study found that different baits had a relatively small impact on the stress capacity of *S. chuatsi* in muscle, but a greater impact on the gastrointestinal tract, particularly the intestine. This result indicates that special attention should be paid to the intestinal changes of *S. chuatsi* when using CF to cultivate them.

## 5. Conclusions

The successful cultivation with CF is a milestone in the *S. chuatsi* farming industry. This not only conserves land resources for breeding bait fish but also offers comprehensive and ample nutrition for *S. chuatsi*. However, due to the feeding characteristics of *S. chuatsi*, it is necessary to conduct continuous research in this area. This study was the first to explore the changes in *S. chuatsi* when fed with CF and LF after transportation stimulation. The findings revealed that when raised with CF, *S. chuatsi* could retain more energy during transportation and exhibit stronger resistance to microbial stress, but they had weaker antioxidant capacity compared to those fed with LF. Therefore, when developing CF for *S. chuatsi* in the future, we recommend dietary antioxidant supplementation (e.g., vitamin E, selenium) as a direction for applied research in future CF formulations.

## Figures and Tables

**Table 1:** Changes in stress indicators and metabolic substances in the blood of *S. chuatsi* under CF and LF.

Indicators	CF	LF
ACP (μmol/min/mL)	0.03 ± 0.00	0.03 ± 0.00
AKP (μmol/min/mL)	0.03 ± 0.00	0.03 ± 0.00
LDH (nmol/min/mL)	13.89 ± 1.15 **	20.68 ± 1.05
T-AOC (μmol Trolox/mL)	0.20 ± 0.01	0.20 ± 0.01
CAT (μmol/min/mL)	30.62 ± 1.81 **	45.63 ± 0.67
SOD (U/mL)	11.84 ± 0.88 **	20.96 ± 0.97
MDA (nmol/mL)	5.87 ± 0.17 **	3.52 ± 0.17
LZM (μg/mL)	6.53 ± 0.42 **	5.20 ± 0.11
TGs (mmol/L)	1.16 ± 0.06 **	0.93 ± 0.05
Glucose (mmol/L)	4.54 ± 0.10 *	3.68 ± 0.35

Note: CF represents the *S. chuatsi* fed with compound feed; LF represents the *S. chuatsi* fed with live bait; * and ** represent *p* < 0.05 and *p* < 0.01, respectively; *n* = 6.

## Data Availability

The datasets used and/or analyzed during the current study are available from the corresponding author upon reasonable request.

## Supplementary Materials

The following supporting information can be downloaded at https://www.mdpi.com/article/10.3390/ani15142154/s1: Table S1 Water quality parameters before and after transportation. Table S2 Stress-related indicators in solid tissues of *Siniperca chuatsi* fed with compound feed and live bait before transportation. Table S3 Stress-related indicators in the blood of *S. chuatsi* fed with compound feed and live bait before transportation. Table S4 The growth rates of stress-related indicators in various tissues of the *S. chuatsi* fed with compound feed and live bait after transportation.
