# Supplementary material for: Stress Response of Siniperca chuatsi to Transport Stimuli Using Compound Feed and Live Bait
Source: Animals (Basel). 2025 Jul 21;15(14):2154. doi: 10.3390/ani15142154 (PMC12291635; doi:10.3390/ani15142154)
Supplement: Supplementary file 1 [file animals-15-02154-s001.zip › animals-3715316-supplementary.pdf]

Table S1 Water quality parameters before and after transportation.

Quality Parameter	$Transport_{pre}$	$Transport_{post}$
T (℃)	15.10 ±0.10	15.30±0.20
DO (mg/L)	7.52±0.30	7.48±0.42
TN (mg/L)	1.33±0.21	1.34±0.43
TP (mg/L)	0.06±0.01	0.06±0.02
NH_4^- (mg/L)	0.11±0.02	0.12±0.01
NO_2^- (mg/L)	0.08±0.00	0.08±0.00
PO_4^- (mg/L)	0.03±0.00	0.03±0.00

Note: $Transport_{pre}$ and $Transport_{post}$ represent before and after transportation, respectively.

Table S2 Stress-related indicators in solid tissues of *Siniperca chuatsi* fed with compound feed and live bait before transportation.

Tissue	ACP (μmol/min/g)		AKP (μmol/min/g)		LDH (nmol/min/g)		T-AOC (μmol Trolox/g)		CAT (μmol/min/g)		SOD (U/g)		MDA (nmol/g)		LZM (μg/g)	
	CF	LF	CF	LF	CF	LF	CF	LF	CF	LF	CF	LF	CF	LF	CF	LF
Brain	0.17±0.00	0.17±0.00	0.18±0.00	0.17±0.00	94.10±7.68	95.96±2.28	1.59±0.06	1.53±0.03	252.89±11.13	261.94±13.04	73.53±5.05	80.88±2.56	25.97±2.78*	23.66±2.38	24.97±1.18	24.83±0.98
Liver	0.22±0.00*	0.21±0.00	0.23±0.00	0.23±0.00	85.68±6.33*	96.49±6.15	1.48±0.04	1.52±0.02	241.16±9.00	235.98±6.90	86.66±5.87	82.14±4.73	20.59±0.78*	18.13±1.66	26.33±0.67	27.61±1.12
Kidney	0.20±0.00**	0.18±0.00	0.22±0.00*	0.19±0.00	66.28±3.85	66.15±6.06	1.43±0.06	1.47±0.07	206.27±7.60	211.59±12.48	37.73±3.65	40.41±6.47	16.77±0.22	16.10±0.64	26.66±1.13	25.87±1.24
Muscle	0.22±0.00**	0.21±0.00	0.26±0.00*	0.22±0.00	87.27±7.52	87.52±3.67	1.50±0.03	1.46±0.05	193.64±6.83	203.26±5.36	42.29±5.30	41.99±3.78	16.07±0.99	15.72±0.85	29.80±1.17	30.89±2.09
Stomach	0.20±0.00*	0.20±0.00	0.21±0.01	0.19±0.01	70.28±5.15	73.97±5.07	1.35±0.05	1.42±0.03	180.07±4.95*	197.43±7.39	42.22±3.22	45.27±5.10	14.27±0.33	13.88±0.57	31.16±1.93	31.33±1.22
Pyloric caecum	0.21±0.00**	0.17±0.00	0.23±0.00	0.23±0.00	74.69±4.17	82.16±4.25	1.36±0.03	1.38±0.07	186.30±8.85	186.14±9.45	43.49±2.63	45.09±5.98	13.67±0.55	14.01±0.36	31.05±2.64	29.53±0.83
Intestine	0.22±0.00**	0.20±0.00	0.22±0.02	0.20±0.01	97.05±6.97	100.75±2.36	1.42±0.04	1.42±0.02	176.27±3.10	187.81±8.79	42.52±5.62	44.54±4.52	13.52±0.32	13.25±0.31	28.85±1.62	29.08±1.23

Note: CF represents the *S. chuatsi* fed with compound feed; LF represents the *S. chuatsi* fed with live bait; n = 6.

Table S3 Stress-related indicators in the blood of *S. chuatsi* fed with compound feed and live bait before transportation.

Enzymes or Metabolites	CF	LF
ACP (μmol/min/mL)	0.02±0.00	0.02±0.00

AKP (μmol/min/mL)	0.03±0.00	0.02±0.00
LHD (nmol/min/mL)	10.79±0.59	11.47±1.20
T-AOC (μmol Trolox/mL)	0.15±0.00	0.15±0.01
CAT (μmol/min/mL)	15.87±0.58	15.58±0.38
SOD (U/mL)	8.26±0.43	8.76±0.23
MDA (nmol/mL)	2.12±0.19	2.28±0.16
LZM (μg/mL)	2.33±0.23	2.52±0.34
TG (mmol/L)	1.42±0.03	1.46±0.08
Glucose (mmol/L)	5.38±0.22	5.38±0.12

Note: CF represents the *S. chuatsi* fed with compound feed; LF represents the *S. chuatsi* fed with live bait; n = 6.

Table S4 The growth rates of stress-related indicators in various tissues of the *S. chuatsi* fed with compound feed and live bait after transportation.

Tissue	ACP		AKP		LDH		T-AOC		CAT		SOD		MDA		LZM		TG		Glucose	
	CF	LF	CF	LF	CF	LF	CF	LF	CF	LF	CF	LF	CF	LF	CF	LF	CF	LF	CF	LF
Brain	26.97%	10.13%	17.38%	14.58%	30.78%	89.08%	11.61%	36.23%	27.48%	55.52%	33.91%	165.42%	102.35%	31.37%	99.87%	59.43%	N/A	N/A	N/A	N/A
Liver	22.83%	15.70%	23.79%	10.81%	41.06%	106.36%	23.51%	30.59%	18.90%	44.81%	42.54%	57.13%	144.94%	80.22%	168.00%	123.50%	N/A	N/A	N/A	N/A
Kidney	30.45%	7.57%	18.01%	5.23%	63.75%	71.44%	14.07%	26.08%	31.88%	57.84%	45.07%	163.75%	127.40%	58.18%	131.59%	106.50%	N/A	N/A	N/A	N/A
Muscle	27.69%	22.31%	15.37%	10.43%	53.25%	77.59%	22.39%	37.85%	54.41%	49.69%	123.51%	148.21%	228.57%	94.08%	92.03%	78.76%	N/A	N/A	N/A	N/A
Stomach	14.32%	8.98%	23.23%	12.56%	59.30%	80.28%	17.97%	46.24%	27.42%	57.07%	50.45%	179.18%	215.63%	37.39%	88.70%	51.22%	N/A	N/A	N/A	N/A
Pyloric caecum	29.36%	9.33%	14.63%	7.60%	36.48%	92.33%	22.25%	34.61%	57.20%	67.23%	80.50%	152.61%	163.45%	135.01%	149.51%	56.50%	N/A	N/A	N/A	N/A
Intestine	32.71%	21.31%	25.27%	9.60%	48.81%	62.23%	11.97%	37.67%	63.69%	75.75%	84.69%	128.62%	203.22%	122.69%	169.02%	53.21%	N/A	N/A	N/A	N/A
Blood	19.24%	19.25%	18.21%	19.16%	28.63%	80.23%	30.59%	29.84%	92.88%	192.94%	43.32%	139.39%	177.53%	54.48%	180.86%	105.83%	-18.15%	-35.79%	-15.74%	-31.61%
Average	25.45%	14.32%	19.49%	11.25%	45.26%	82.44%	19.30%	34.89%	46.73%	75.11%	63.00%	141.79%	170.39%	76.68%	134.95%	79.37%	-18.15%	-35.79%	-15.74%	-31.61%

Note: The value was obtained by comparing it after transportation with before transportation. CF represents the *S. chuatsi* fed with compound feed; LF represents the *S. chuatsi* fed with live bait; "N/A" represents no relevant data; growth rate (%) = ($transport_{post}$ - $transport_{pre}$)/ $transport_{pre}$ × 100; n = 6.

Formulae for calculating enzyme activity:

ACP (μmol/min/g) = 0.1 * (OD*a* - OD*b* + 0.0018) / W * D

ACP (μmol/min/mL) = 0.1 * (OD*a* - OD*b* + 0.0018) * D

Note: OD*a* represents the absorbance value of the sample. OD*b* represents the absorbance value of the blank control. W represents the sample weight. D represents the sample dilution ratio.

AKP (μmol/min/g) = 0.128 * (OD*a* - OD*b* - 0.0019) / W * D

AKP (μmol/min/mL) = 0.128 * (OD*a* - OD*b* - 0.0019) / * D

Note: OD*a* represents the absorbance value of the sample. OD*b* represents the absorbance value of the blank control. W represents the sample weight. D represents the sample dilution ratio.

LDH (nmol/min/g) = 98.9 * (OD*a* - OD*b*) / W * D

LDH (nmol/min/mL) = 98.9 * (OD*a* - OD*b*) / * D

Note: OD*a* represents the absorbance value of the sample at "n + 1" mins. OD*b* represents the absorbance value of the sample at "n" mins. W represents the sample weight. D represents the sample dilution ratio.

T-AOC (μmol Trolox/g) = 0.57 * (OD*a* - OD*b* + 0.0218) / W * D

T-AOC (μmol Trolox/mL) = 0.57 * (OD*a* - OD*b* + 0.0218) / * D

Note: OD*a* represents the absorbance value of the sample. OD*b* represents the absorbance value of the blank control. W represents the sample weight. D represents the sample dilution ratio.

CAT (μmoL/min/g) = 141.6 * (OD*a* - OD*b* + 0.0137) / W * D

CAT (μmoL/min/mL) = 141.6 * (OD*a* - OD*b* + 0.0137) / * D

Note: OD*a* represents the absorbance value of the blank control. OD*b* represents the absorbance value of the sample. W represents the sample weight. D represents the sample dilution ratio.

$SOD\ (U/g) = 10 * A / (1- A) / W *D; A\ (\%) = [(OD_c - OD_d) - (OD_a - OD_b)]/ (OD_c - OD_d) * 100\%$

$SOD\ (U/mL) = 10 * A / (1- A) / *D; A\ (\%) = [(OD_c - OD_d) - (OD_a - OD_b)]/ (OD_c - OD_d) * 100\%$

Note: A represents the inhibition rate of SOD. W represents the sample weight. D represents the sample dilution ratio. OD*a* represents the absorbance value of the sample. OD*b* represents the absorbance value of the blank in the experimental group. OD*c* represents the absorbance value of the control group. OD*d* represents the absorbance value of the blank in control group.

$MDA\ (nmol/g) = 32.3 * (OD_a - OD_b) / W * D$

$MDA\ (nmol/mL) = 32.3 * (OD_a - OD_b) / * D$

Note: OD*a* represents the absorbance value of the sample in 532 nm. OD*b* represents the absorbance value of the sample in 600 nm. W represents the sample weight. D represents the sample dilution ratio.

$LZM\ (\mu g/g) = 10 * (OD_a - OD_b) / (OD_c - OD_d) /W * D$

$LZM\ (\mu g/mL) = 10 * (OD_a - OD_b) / (OD_c - OD_d) * D$

Note: OD*a* represents the absorbance value of the sample at "n + 2.5" mins. OD*b* represents the absorbance value of the sample at "n" mins. OD*c* represents the absorbance value of the standard sample at "n + 2.5" mins. OD*d* represents the absorbance value of the standard sample at "n" mins. W represents the sample weight. D represents the sample dilution ratio.

$TG\ (mmol/L) = 0.782 * (OD_a - OD_b) / (OD_c - OD_b) * D$

Note: OD*a* represents the absorbance value of the sample. OD*b* represents the absorbance value of the blank control. OD*c* represents the absorbance value of the standard sample. D represents the sample dilution ratio.

$Glucose\ (mmol/L) = 5.55 * OD_a / OD_b * D$

Note: OD*a* represents the absorbance value of the sample. OD*b* represents the absorbance value of the standard sample. D represents the sample dilution ratio.
